# Accurate control of the covalent functionalization of single-walled carbon nanotubes for the electro-enzymatically controlled oxidation of biomolecules

**DOI:** 10.3762/bjnano.9.257

**Published:** 2018-10-26

**Authors:** Naoual Allali, Veronika Urbanova, Mathieu Etienne, Xavier Devaux, Martine Mallet, Brigitte Vigolo, Jean-Joseph Adjizian, Chris P Ewels, Sven Oberg, Alexander V Soldatov, Edward McRae, Yves Fort, Manuel Dossot, Victor Mamane

**Affiliations:** 1 LCPME, UMR CNRS-Université de Lorraine 7564, 405 rue de Vandoeuvre, F-54602 Villers-lès-Nancy, France; 2 SRSMC, UMR CNRS-Université de Lorraine 7565, Campus Victor Grignard, Faculté des Sciences et Technologies, F-54506 Vandoeuvre-lès-Nancy, France; 3 Department of Engineering Sciences and Mathematics, Lulea Technical University, Sweden; 4 IJL, UMR CNRS-Université de Lorraine 7198, Parc de Saurupt - CS 50840, 54011 Nancy Cedex, France; 5 IJL, UMR CNRS-Université de Lorraine 7198, Campus Victor Grignard, Faculté des Sciences et Technologies, 54506 Vandoeuvre-lès-Nancy Cedex, France; 6 IMN, UMR CNRS-Université de Nantes 6502; 7 New address: Institut de Chimie de Strasbourg, UMR CNRS-Université de Strasbourg 7177

**Keywords:** biosensing, carbon nanotubes, covalent functionalization, electrocatalysis, ferrocene

## Abstract

Single-walled carbon nanotubes (SWCNTs) were functionalized by ferrocene through ethyleneglycol chains of different lengths (FcETGn) and the functionalized SWCNTs (f-SWCNTs) were characterized by different complementary analytical techniques. In particular, high-resolution scanning electron transmission microscopy (HRSTEM) and electron energy loss spectroscopy (EELS) analyses support that the outer tubes of the carbon-nanotube bundles were covalently grafted with FcETGn groups. This result confirms that the electrocatalytic effect observed during the oxidation of the reduced form of nicotinamide adenine dinucleotide (NADH) co-factor by the f-SWCNTs is due to the presence of grafted ferrocene derivatives playing the role of a mediator. This work clearly proves that residual impurities present in our SWCNT sample (below 5 wt. %) play no role in the electrocatalytic oxidation of NADH. Moreover, molecular dynamic simulations confirm the essential role of the PEG linker in the efficiency of the bioelectrochemical device in water, due to the favorable interaction between the ETG units and water molecules that prevents π-stacking of the ferrocene unit on the surface of the CNTs. This system can be applied to biosensing, as exemplified for glucose detection. The well-controlled and well-characterized functionalization of essentially clean SWCNTs enabled us to establish the maximum level of impurity content, below which the f-SWCNT intrinsic electrochemical activity is not jeopardized.

## Introduction

Carbon nanotubes (CNTs) have been recognized as interesting candidates for developing electrochemical sensors for almost two decades [1–3]. They have been used to modify electrodes (e.g., glassy carbon electrodes, GCEs) in order to decrease the overpotential value, increase sensitivity and reduce the occurrence of electrode fouling by degradation of the analyzed (bio)molecules [4]. They can increase the electron transfer rate between electrode and target molecules and decrease electrode response time [3,5]. We and others have reported some interesting electrocatalytic effects when using CNT-modified GCEs for the voltamperometric detection of (bio)molecules [6–14].

Two major hurdles have to be tackled when designing CNT-modified GCEs as biosensors: the water solubility and the cleanliness of employed CNTs in order to avoid misinterpreted results.

The solubility of CNTs in water, a usual solvent for biosensors, is quite low. The strategies used to increase their water solubility have been either i) to chemically modify them by putting oxidative defects on their sidewalls and extremities to decrease their hydrophobicity, ii) to use non-covalent interaction with hydrophilic biomolecules or surfactants [15–18] or iii) to use covalently grafted polyethyleneglycol (PEG) linkers, as described by us [9,11–12].

Many studies use multiwall CNTs (MWCNTs) as a rather cheap source of CNTs. They are often mixed with an electron mediator that interacts non-covalently with the CNT sidewalls. The electron mediator is used as an electron shuttle towards biologically relevant molecules, such as enzymes or co-factors, especially nicotinamide adenine dinucleotide hydride (NADH) [6,8,13,19]. The CNTs and mediator are co-deposited on the GCE using a polymer. Chitosan is often used as a cheap biodegradable biopolymer with good compatibility with CNTs for making adequate suspensions before deposition on the GCE [20]. The main problem arising from the non-covalent interaction between CNTs and the electron mediator is that the latter may diffuse inside the analyzed medium, resulting in a progressive decrease of the sensor efficiency. A good alternative is therefore to covalently graft an electron mediator onto the CNT sidewalls. While CNT chemistry is now well developed, it can still remain somewhat challenging depending on the quality of the nanotube sample. Indeed, if the sample contains many carbonaceous impurities, these can also be functionalized and contribute to the final electrochemical signal. Several studies have underlined the role of residual metallic and carbonaceous impurities present in CNT samples on the electrocatalytic effects often reported in the literature [21–26]. The presence of such impurities may modify the reactivity of the CNTs themselves and can make it difficult to qualify and quantify the covalent functionalization of the CNTs. If one wants to control the chemistry made on the tubes at each step, it is absolutely mandatory to start from a very clean sample. Purified HiPco^®^ SWCNTs are now commercially available at reasonable prices and constitute such a clean sample.

In our previous work, we have described the synthesis of covalently functionalized HiPco SWCNTs (f-SWCNTs) with ferrocene through PEG linkers, which presented good electrochemical efficiency for NADH oxidation [12]. However, two questions remained open regarding: i) the role of the PEG linker, besides its ability to increase the water solubility of f-SWCNTs and ii) the influence of metallic and/or carbonaceous impurities that may be present in the CNT sample.

**Figure 1:**
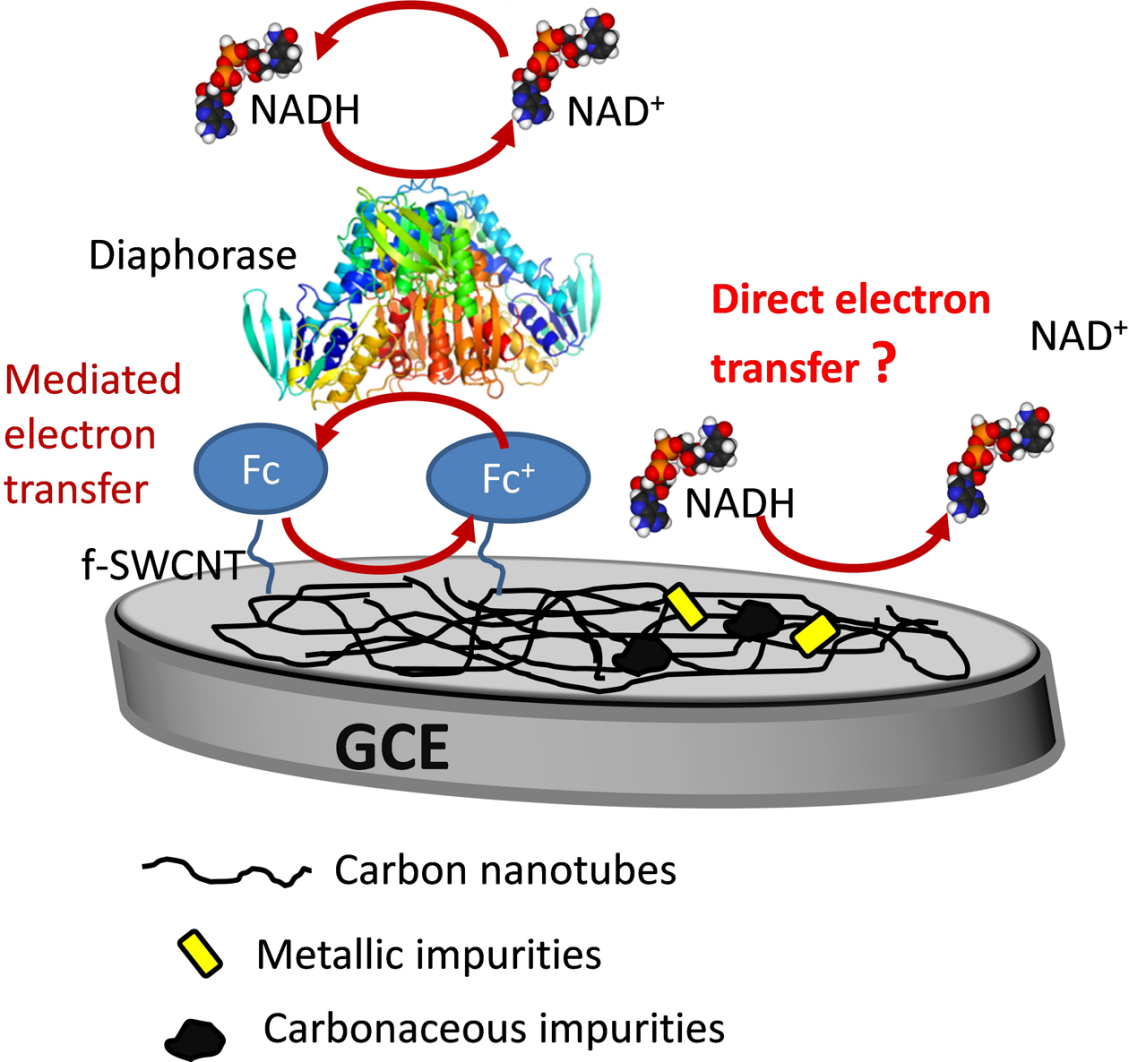
A working electrode (WE) incorporating the f-SWCNTs deposited on the GCE surface.

In this article, by using advanced and complementary analytical techniques, we fully confirm the covalent nature of the chemical grafting. In particular, high-resolution scanning electron transmission microscopy (HRSTEM) and electron energy loss spectroscopy (EELS) analyses strongly supports the role of ferrocene in the observed electrocatalytic effect for NADH oxidation and rule out the hypothetic role of metallic and carbonaceous impurities. Moreover, the role of the PEG linker in the good electrochemical response was studied by molecular dynamics, which show that favorable interaction between the ETG units and water molecules prevents π-stacking of the ferrocene unit on the surface of the CNTs, therefore allowing for a good electron transfer. Figure 1 summarizes the context of this study.

## Results and Discussion

### Source of clean SWCNTs and strategy of functionalization

**Figure 2:**
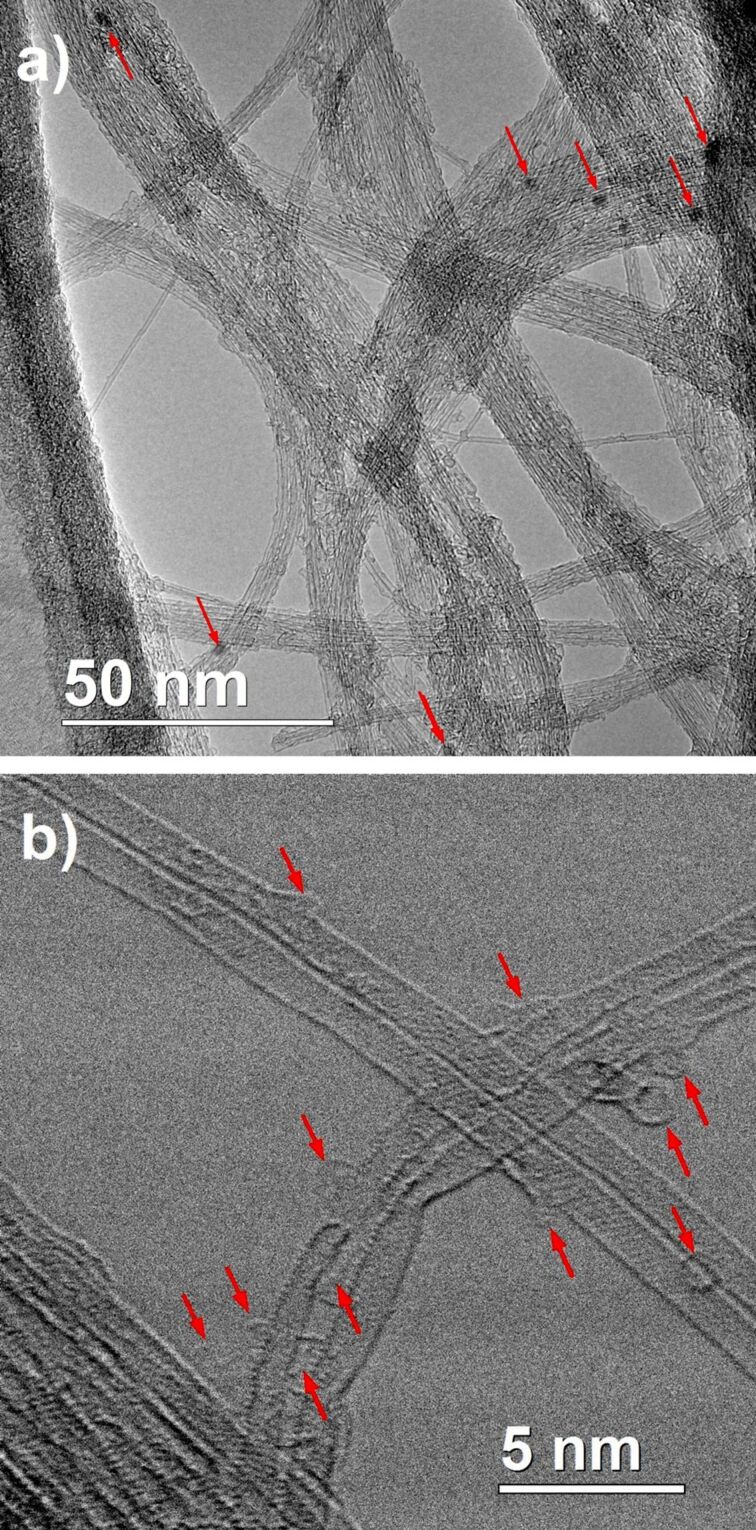
a) HRTEM micrographs of the raw HIPCO material. The arrows point out residual iron nanoparticles. b) HRSTEM BF image showing carbonaceous impurities at the surface of SWCNTs.

A HiPco SWCNT sample was purchased from Nanointegris Inc. of the purest grade (“super purified grade”, less than 5% of residual catalytic particles, http://www.nanointegris.com/en/hipco, accessed August 2016). Figure 2a gives an example of the HRTEM image of this starting material. A small amount of residual iron catalyst is visible (dark particles pointed out by red arrows). Carbonaceous impurities are mainly present in the form of carbon remains of nanometric size deposited along the CNT sidewalls and on the bundles (see red arrows in HRSTEM BF image presented in Figure 2b) but they are in very small amounts and the sample is clearly quite clean. In the rest of the text, to avoid the use of the commercial term HiPco, the symbol of our samples will be written HIPCO.

**Figure 3:**
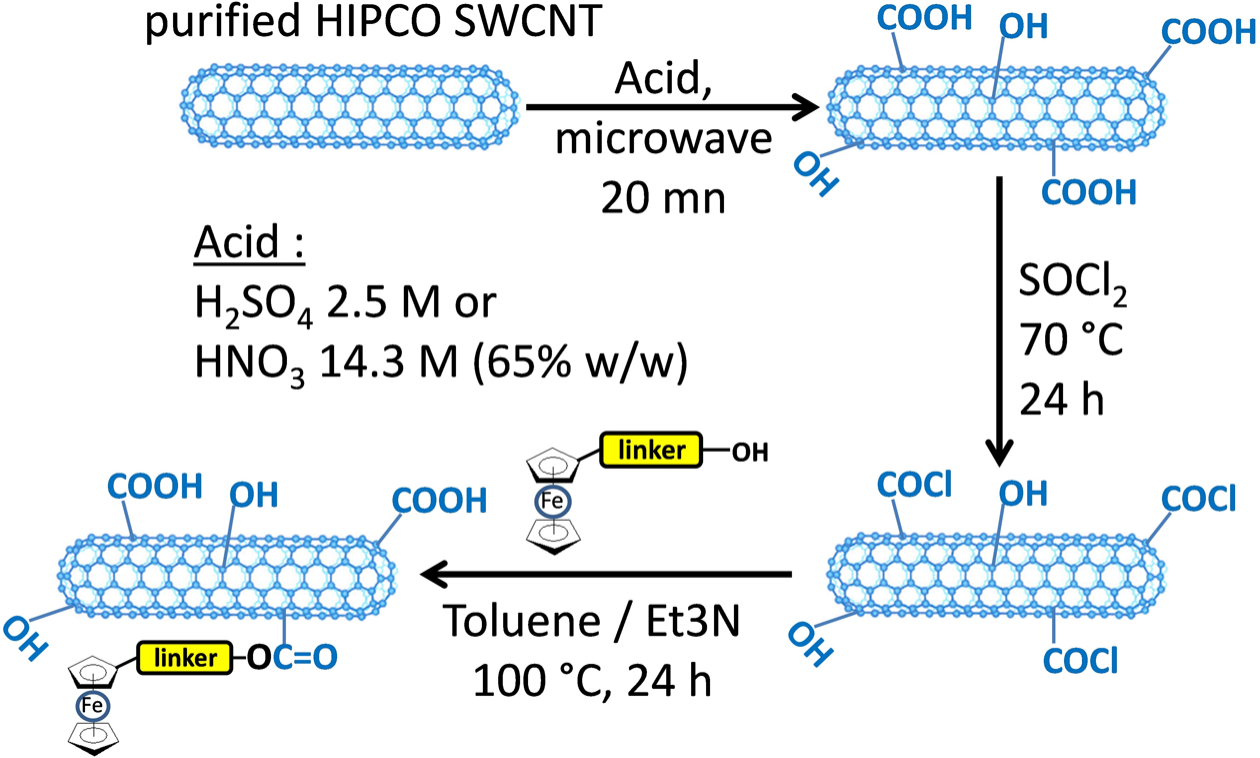
Three-step covalent functionalization of HIPCO SWCNTs using different acidic conditions for step 1.

The SWCNTs were oxidized in acidic media using microwave irradiation to control the number of oxidized groups created on the CNT sidewalls [9,11]. Several microwave irradiation times were investigated but an optimum was found at around 20 min. Two different acidic media were tested: a quite conventional concentrated HNO_3_ solution (65% w/w) and a rather diluted H_2_SO_4_ solution (2.5 mol·L^−1^). The latter medium is greener and safer than a concentrated HNO_3_ solution. Reaction with SOCl_2_ was further realized and then ferrocene moieties coupled to linkers were added to the chlorinated CNTs for covalently attaching the ferrocene electron shuttle to the nanotube sidewalls. The principle of our strategy is indicated in Figure 3.

Oxidation of SWCNTs using concentrated HNO_3_ (65% w/w, 14.3 M) or 2.5 M H_2_SO_4_, and subsequent treatment with SOCl_2_ were realized following the previously described protocols [9,27–28]. We obtained COCl-SWCNT samples that were then used to covalently graft ferrocene derivatives on the CNT sidewalls.

### Oxidation and chlorination steps

Figure 3 indicates that the first step needed to subsequently graft ferrocene moieties onto SWCNTs is to create oxidative defects on their sidewalls. In the literature, this is often done by heating under reflux the CNT sample in a strong acidic medium for several hours or days. Doing so, many oxidized functions are introduced but the nanotubes are also cut and shortened. Since we intended to retain the essential electronic properties of the CNTs in the aim of making an electrochemical biosensor, we performed the oxidation step using microwave irradiation. Microwaves indeed promote oxidation through a fast thermal activation, which enables performing the oxidation step in only a few minutes and allows one to roughly control the number of defects introduced by tuning the irradiation time. A few milligrams of the corresponding oxidized SWCNTs were analyzed in each case before proceeding to step 2. At this stage, the samples were protected under argon gas to avoid any moisture contamination and directly analyzed by HRTEM, XPS and TGA-MS. Once opened to air, the samples were analyzed by Raman spectroscopy.

### Functionalization by ferrocene derivatives

**Figure 4:**
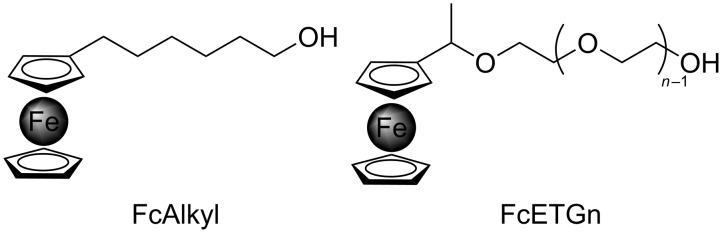
Ferrocene derivatives (FcAlkyl and FcETG*_n_*) with different linkers.

Ferrocene derivatives were grafted onto CNT sidewalls by reacting the alcohol group of the ferrocene linkers to the COCl groups present after step 2 on the CNT sidewalls. We investigated several linkers, in order to see if the chemical nature of the linker and/or its length might influence the electrochemical response of our final device. An alkyl chain and polyethylene glycol linkers with various chain lengths were used in this study (Figure 4). The corresponding ferrocene derivatives, FcAlkyl and FcETGn, were prepared according to known procedures [29–30].

Nomenclature of the samples will indicate the oxidation process (HNO_3_ or H_2_SO_4_) and for grafted samples, the nature of the grafted group (FcAlkyl or FcETG*_n_*). For instance, HIPCO-HNO_3_-FcETG_8_ means that the SWCNTs were oxidized in HNO_3_ 65% and grafted with the FcETG_8_ ferrocene derivative.

**Figure 5:**
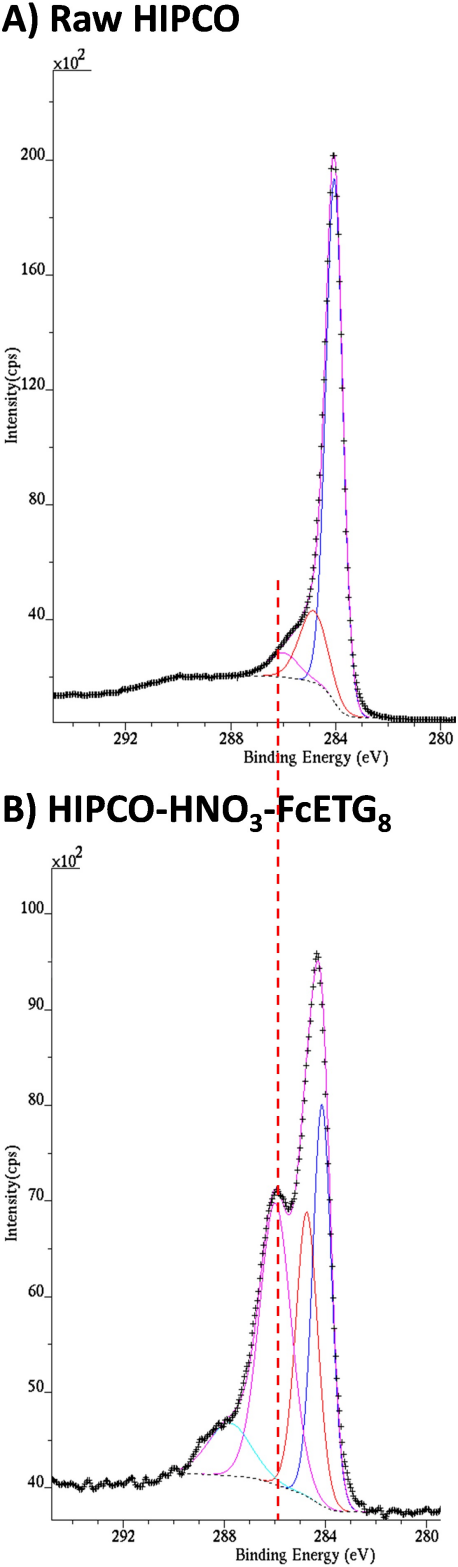
Detailed C 1s spectra of A) raw HIPCO and B) HIPCO-HNO_3_-FcETG_8_. The binding energies of the Fe 2p components were always at 720.4 eV and 706.5 eV for the 2p_1/2_ and 2p_3/2_ components, respectively, which are typical values for the Fe(II) oxidation state in ferrocene compounds [31].

After step 3 of the functionalization process, we used a set of complementary techniques to determine the success of the covalent functionalization of the CNT samples. XPS analyses were realized on CNT powders to see if the ferrocene groups were indeed present at the surface of the bundles, using the Fe 2p signal as a marker of the grafted groups (Figure S1A, Supporting Information File 1). Table 1 reports the atomic percentages found on raw, oxidized and functionalized HIPCO samples, as well as the spectral components of the C 1s signal obtained by spectral decomposition using four components for the oxidized/functionalized samples. The component at 284 eV is attributed to sp^2^-hybridized carbon atoms, while that at 284.6–284.8 eV is assigned to sp^3^-hybridized carbon atoms. Oxidized carbon atoms gave two signals at 286 eV (probably C–OH or ether groups) and 288 eV (lactone or COOH groups). Since these two peaks correspond to oxygenated functions, their areas have been added and Table 1 reports the sum of these two components. Two examples of spectral decomposition are reported in Figure 5, one for the raw HIPCO sample (Figure 5A) and one for HIPCO-HNO_3_-FcETG_8_ (Figure 5B).

**Table 1:** Atomic percentages and C 1s contributions deduced from XPS analyses of raw, oxidized and functionalized samples.

sample	atomic concentrations						
	atom % Fe	atom % O	atom % C	atom % O/ atom % C	atom % C sp^2^ (284 eV)	atom % C sp^3^ (284.6–284.8 eV)	atom % C (C–O/C=O) (286/288 eV)

HIPCO Raw	0	6.6	86.3	0.076	75	17.3	7.7
HIPCO-HNO_3_	0	13.7	84	0.163	68.6	16.7	14.7
HIPCO-HNO_3_-FcAlkyl	1.3	13.9	84.4	0.165	56.2	23	20.8
HIPCO-HNO_3_-FcETG_2_	1.9	14.4	62	0.232	72.3	14.1	13.6
HIPCO-HNO_3_-FcETG_8_	0.8	19.1	59.6	0.32	29.9	25	45.1
HIPCO-H_2_SO_4_	0	10.4	75	0.139	70.4	14.8	14.8
HIPCO-H_2_SO_4_-FcETG_2_	1.8	15.4	63.1	0.244	69.8	10.1	20.1
HIPCO-H_2_SO_4_-FcETG_5_	1.2	16.5	69.5	0.237	60.1	18.4	21.5

After oxidizing SWCNTs under acidic conditions under microwave irradiation, the atomic ratio of oxygen to carbon (%O/%C) is increased for both HNO_3_ and H_2_SO_4_ conditions. Furthermore, the number of oxygenated functions, quantified by the percentage of the C–O/C=O components contributing to the C 1s signal, has also doubled. Oxidation step 1 has therefore increased the number of oxidized defects on CNT sidewalls and extremities, at least at the surface of the bundles, which was probed by XPS. It should be pointed out that our raw HIPCO sample has already been submitted to unknown chemical treatments by Nanointegris to reach the claimed degree of purity indicated on their website. It is therefore not surprising to already find oxygenated functions in the raw sample, these defects having certainly been introduced by the chemical treatment.

We also underline the fact that no iron or other metals are visible by XPS spectroscopy, while TEM micrographs have shown some residual catalyst particles embedded in carbonaceous remains or inside CNT bundles, as shown in Figure 2a. These carbon shells certainly hide the metallic species from the XPS analysis, which is essentially a surface-specific method. The clear Fe 2p signal obtained for functionalized samples and reported for instance in Figure S1B (Supporting Information File 1) for the HIPCO-FcETG_2_ sample arises exclusively from the grafted groups. The binding energies of the Fe 2p components were always at 720.4 eV and 706.5 eV for 2p_1/2_ and 2p_3/2_ components, respectively, which are typical values for the Fe(II) oxidation state in ferrocene compounds [31].

Table 1 indicates that around 1–2 atom % Fe is detected in the samples functionalized with ferrocene derivatives (entries 3, 4, 5, 7 and 8), which is a low level of functionalization. It also reports an increase of the oxygen content after grafting FcETG*_n_* groups and this is particularly true for the HIPCO-HNO_3_-FcETG_8_ sample (entry 5). The change in the detailed C 1s spectrum if compared to that of the starting material is quite obvious, as shown in Figure 5B. The PEG linker is quite long in this sample, and the surface-specific sensitivity of the XPS technique means that the detected signal is dominated by the contribution of the linker. Another interesting observation is that for the HIPCO-HNO_3_-FcAlkyl sample, there is a significant increase of the sp^3^ contribution in the detailed C 1s spectrum, also coming from the linker (entry 3). Therefore, the use of very clean HIPCO samples enables detecting by XPS both the tagging Fe atom of ferrocene derivatives and the contributions of the linkers.

**Figure 6:**
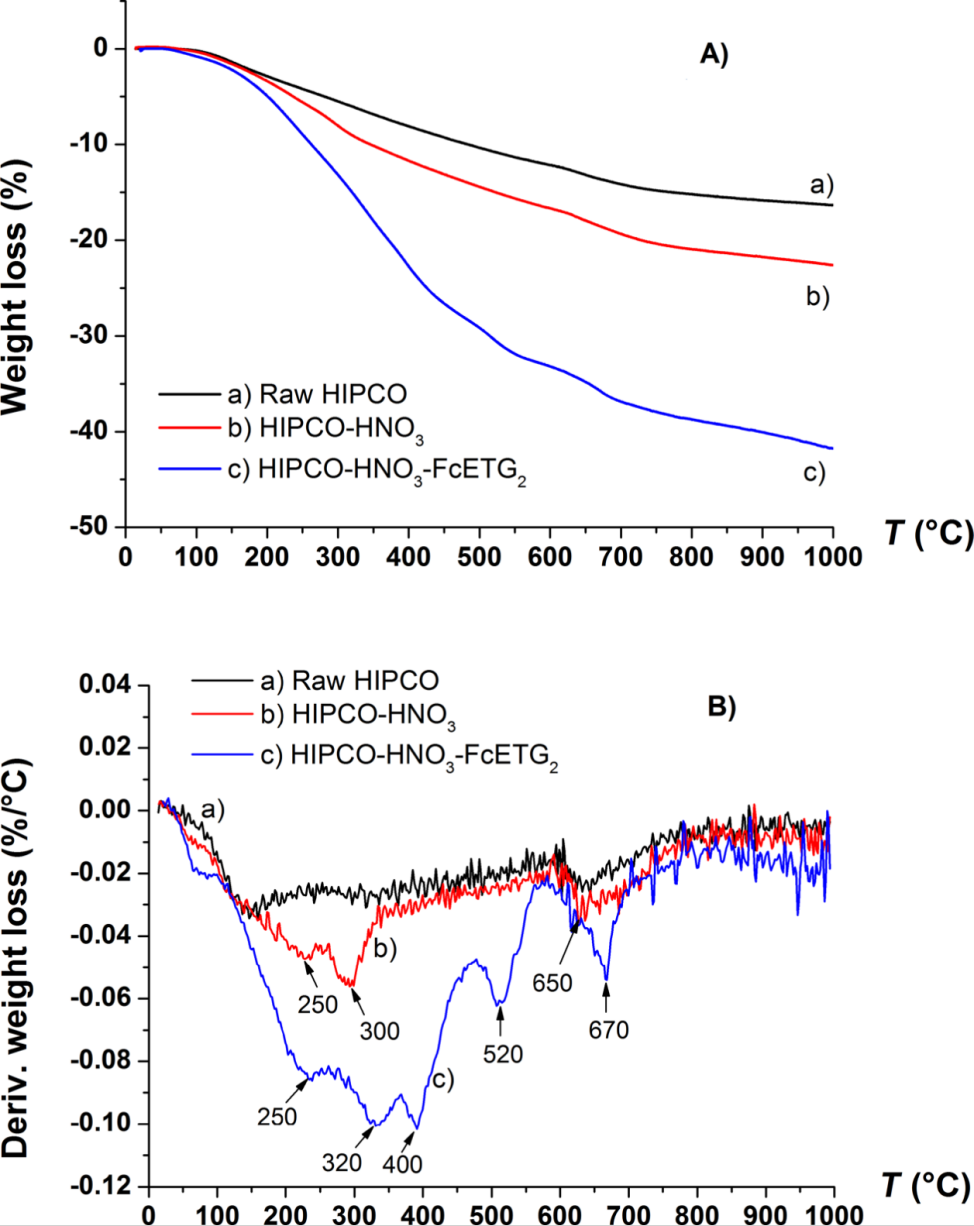
A) TGA curves obtained under He for three samples. B) Derivative curve of the weight loss for the same three samples. Arrows indicate the main peaks in temperature associated with departure of some chemical groups attached to SWCNT sidewalls.

XPS analyses prove the presence of ferrocene derivatives at the surface of CNT bundles, but they cannot confirm the covalent nature of the functionalization process. To do this, we have performed TGA-MS analyses of our samples under an inert gas (He). Figure 6A gives an example of the TGA curves obtained for raw, HIPCO-HNO_3_ and HIPCO-HNO_3_-FcETG_2_ samples.

**Figure 7:**
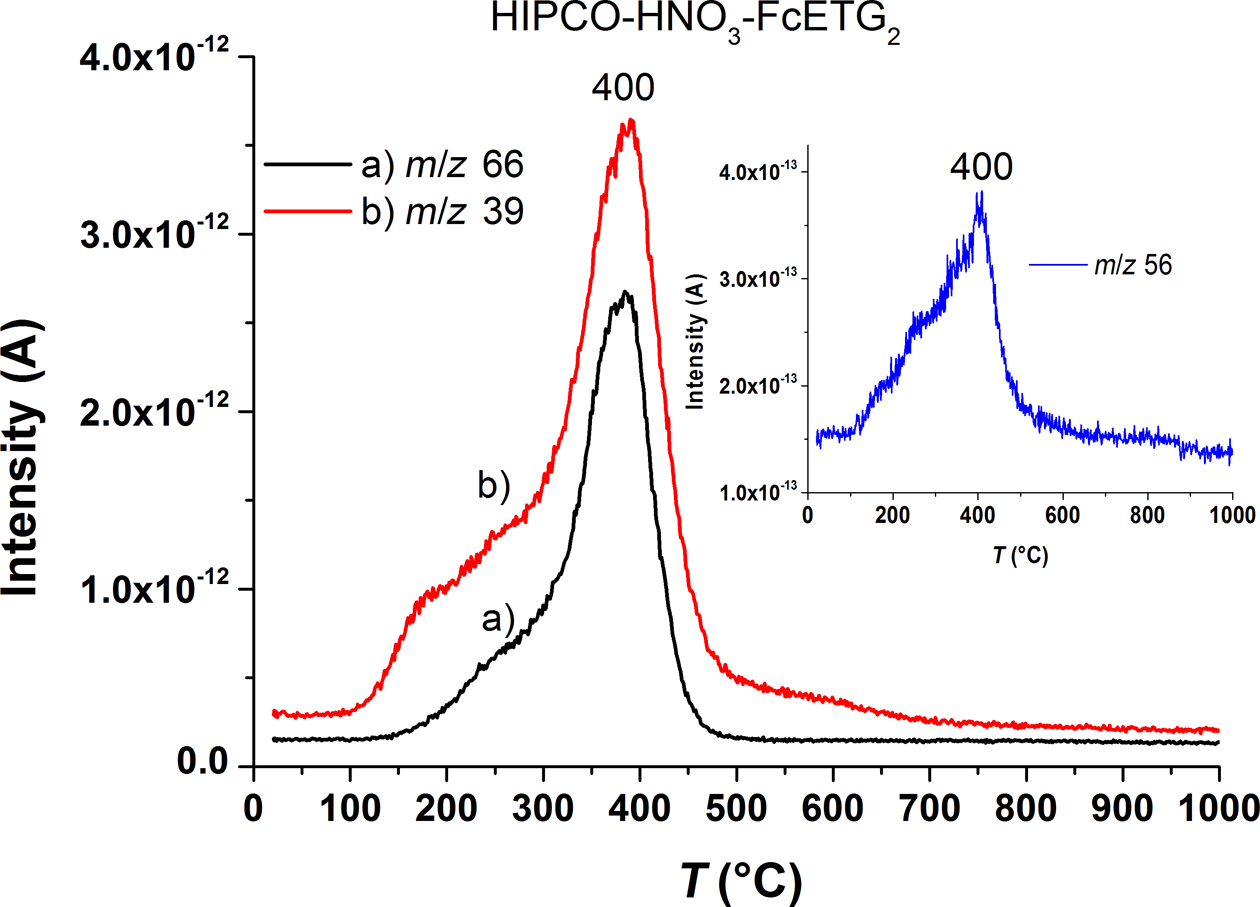
Mass spectrometry channels used to show the departure peaks of the cyclopentadienyl group and its main fragments at m/z = 66 and m/z = 39. Inset: the departure peak of the iron atoms of the ferrocene groups.

The mass spectrometer coupled to the TGA system allowed for the examination of the nature of the departing groups. Compared to the raw sample (curve a), the TGA curve after oxidation clearly shows an increase of weight loss above 300 °C for the oxidized sample due to additional oxygenated functions on the nanotube surface (curve b). A greater increase of weight loss was observed for the HIPCO-HNO_3_-FcETG_2_ sample indicating that FcETG2 functions were covalently grafted (curve c). This is consistent with the greater number of oxidized defects detected by XPS as well as the presence of ferrocene derivatives in the sample. Moreover, cyclopentadienyl groups, which are expected from the decomposition of ferrocenes, can be followed by detecting the main fragments expected for cyclopentadienyl, i.e., at *m*/*z* 66 and at *m*/*z* 39. Figure 7 reports the current intensity corresponding to these fragments, which were evacuated between 150 and 450 °C; the majority being detected around 370–400 °C. The detection of iron ions at *m*/*z* 56 was also found, as shown in the inset of Figure 7.

Figure 7 also indicates that the ferrocene groups are detached mainly above 300 °C, a temperature that is too high to correspond to physisorbed molecules. TGA-MS experiments therefore confirm the covalent grafting of the ferrocene derivatives on the CNTs. Table 2 summarizes the global weight losses and the main departure peaks corresponding to the data plotted in Figure 6.

**Table 2:** Percentages of weight loss and temperature of the main desorption peaks measured in TGA-MS for raw, oxidized and functionalized HIPCO samples.

sample	weight loss (in %) at 1000 °C	temperatures of main departures peaks (°C)

HIPCO raw	16	150, 650
HIPCO-HNO_3_	22	250, 300, 650
HIPCO-HNO_3_-FcETG_2_	42	250–400, 520, 670

Figure S2 (Supporting Information File 1) reports the departure of CO and CO_2_ fragments at *m*/*z* 28 and *m*/*z* 44, respectively, for the HIPCO-HNO_3_ and HIPCO-HNO_3_-FcETG_2_ samples. For HIPCO-HNO_3_, CO_2_ fragments are mainly evacuated between 150 and 500 °C, while a significant loss of CO is visible around 650 °C. These peaks correspond to the evacuation of oxidized functions introduced by the microwave-assisted oxidation process. For the HIPCO-HNO_3_-FcETG_2_ sample, additional peaks around 340 and 675 °C are visible for both CO and CO_2_ fragments. They are due to the fragmentation of the polyethylene glycol linkers, confirming that ferrocene derivatives are covalently attached to CNT sidewalls.

**Figure 8:**
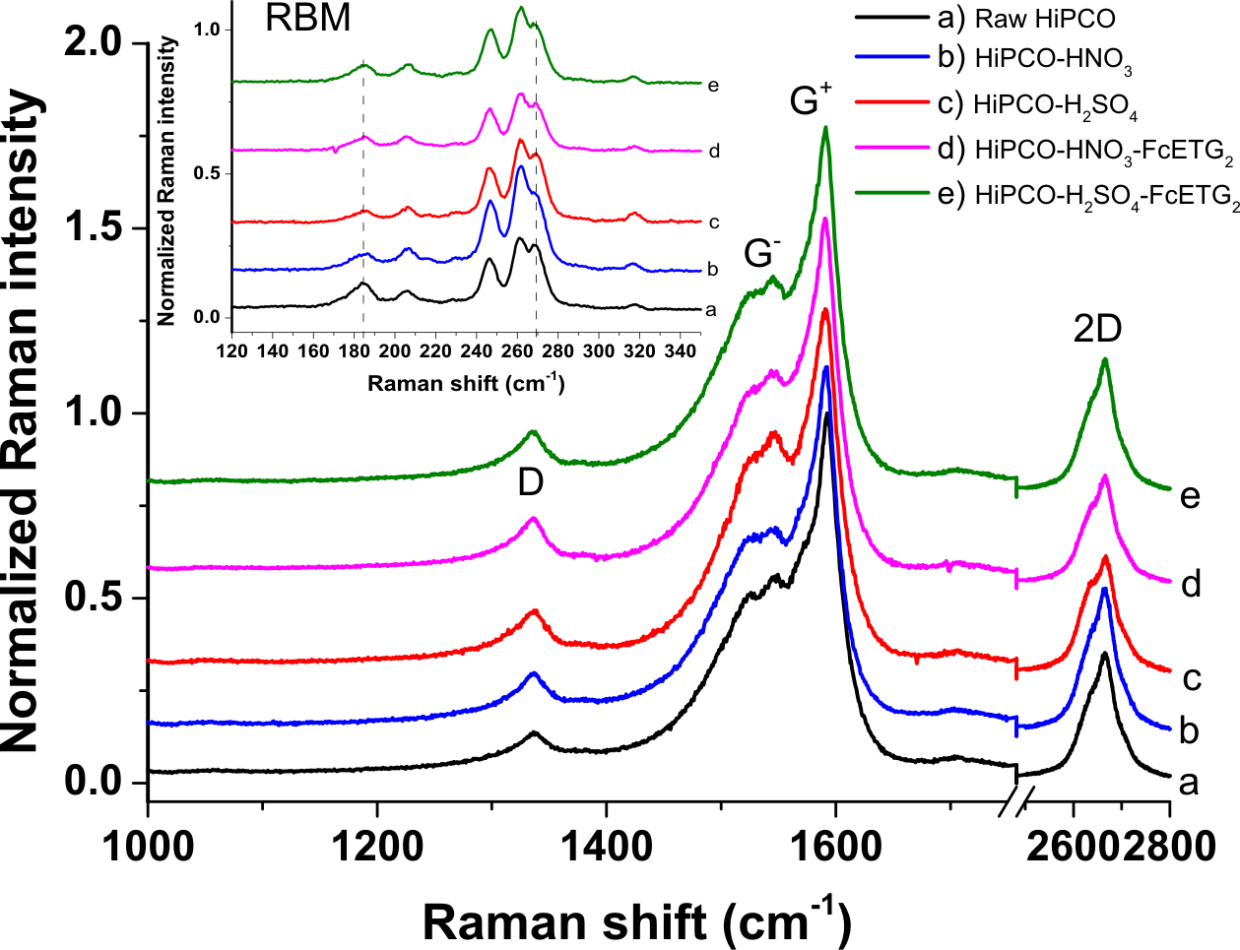
Raman data of raw and f-SWCNTs taken with a laser at 514 nm.

XPS and TGA-MS experiments thus show that oxidized defects can be detected for samples having undergone the oxidation and functionalization steps. Raman spectroscopy is a method of choice to observe the creation of sp^3^ defects in carbon nanostructures, due to the presence in the Raman spectrum of a dispersive defect-induced band, called the D band, around 1350 cm^−1^. Figure 8 reports the Raman spectra of raw, oxidized and functionalized samples using a laser wavelength of 514 nm. In Figure S3 (Supporting Information File 1), some Raman spectra obtained for the samples using a laser at 458 nm are also reported. The data for each sample correspond to the average spectrum obtained from 20 spectra, and they are therefore statistically significant.

Surprisingly, the intensity of the D band at 1350 cm^−1^, associated with the presence of structural defects, remains essentially constant if compared to the G^+^ and G^−^ bands for all samples. The radial breathing modes (RBM) shown in the inset are also barely affected by the chemistry steps. The structural integrity of the CNTs in resonance with the laser energies used in this study (514 and 458 nm) remains quite good. That is, the number of sp^3^ defects that have been created by the chemical processes is low enough to keep the intensity of the D band almost constant. However, XPS and TGA-MS experiments have shown an increased number of oxidized functions, which generally involve the creation of sp^3^-hybridized carbon atoms in the CNT sidewalls. To solve this apparent contradiction, we can make the hypothesis that microwave irradiation in acidic media mainly converts already-existing sp^3^ defects in the raw CNTs into oxidized functions such as alcohol and carboxylic acid functions, but does not introduce many new defects as does the purely thermal process (heating under reflux). Moreover, our functionalization process uses bundles of CNTs, and probably most of the inner tubes within the bundles are not affected by the chemical processes. Raman spectroscopy also probes these inner tubes, which may explain the lack of significant change of the D-band intensity in the spectra. This explanation is indeed coherent with the low value of the Fe atomic percentage detected in XPS (Table 1). We have put a small number of ferrocene functions on the surface of our HIPCO bundles, and the integrity of the carbon structure is essentially preserved. This is a very interesting observation since it validates our global approach of using microwave irradiation rather than more conventional thermal routes. Retaining the structural integrity of CNTs (and therefore their electronic properties) is essential in our case to obtain a good electrochemical signal.

**Figure 9:**
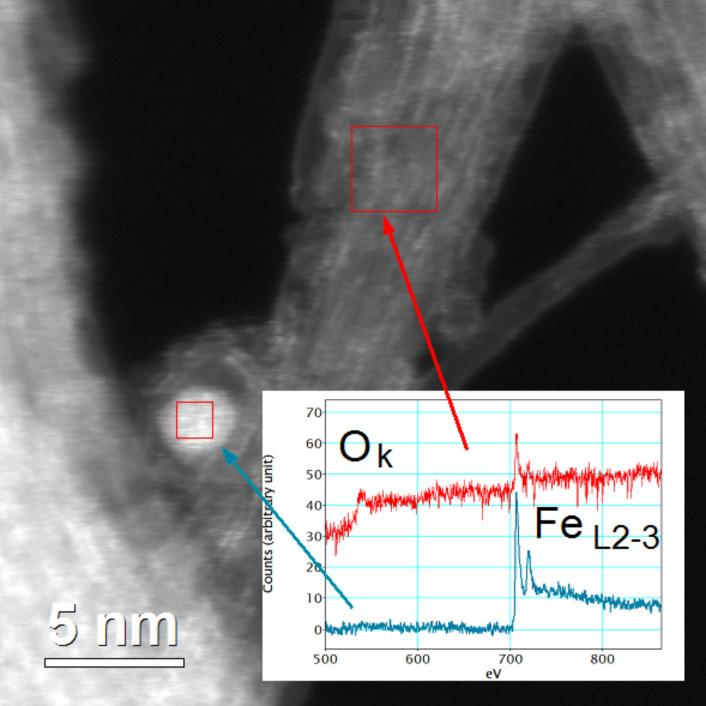
An example of a STEM HAADF image of the HIPCO-HNO_3_-FcETG_2_ sample. Inset: EELS spectra of the catalyst particle (blue arrow) and of the bundle recorded in the area of the red square.

HRSTEM and EELS analyses confirm the presence of ferrocene derivatives on the surface of the bundles. Figure 9 shows a HAADF micrograph of the HIPCO-HNO_3_-FcETG_2_ sample.

In HAADF the intensity of the signal is linked to the average “*Z”* value of the atoms. On the image presented in Figure 9, the zones that contain atoms heavier than carbon appear as white areas and the thickest area also appears brighter. One residual catalyst particle can be easily identified in Figure 9 as a bright spot with a diameter close to 2.5–3.0 nm. On the bundle some small white dots can also be seen. EELS spectra were recorded of the catalytic particle and in the middle of the bundle, in the area where white dots are visible (Figure 9 inset). On the particle, only a Fe_L2-3_ signal could be detected, typical of iron in its metallic state. From the bundle, both O_K_ and Fe_L2-3_ signals are clearly visible. These signals are compatible with the presence of ferrocene groups grafted through ETG linkers.

**Figure 10:**
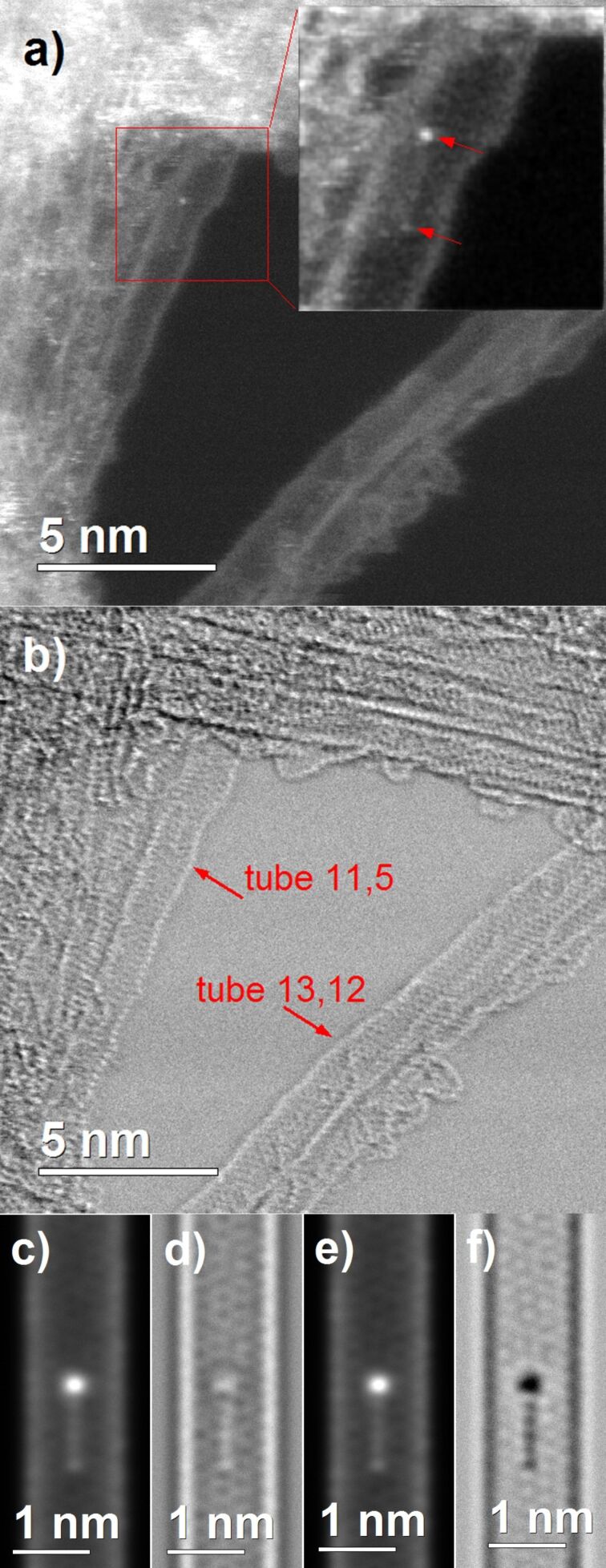
HIPCO-HNO_3_-FcETG_2_ sample analyzed by STEM: a) HAADF image. The inset focuses on the FcETG molecule, the top arrow points to the ferrocene and the bottom arrow to the ester link. b) BF image simultaneously recorded for chiral index assignment. c–f) Simulated images of HAADF (c and e) and BF (d and f) of a 11,6 SWCNT grafted with a FcETG_2_ molecule for over-focus of 1.7 nm (c and d) and under-focus of −6.3 nm.

In the HAADF images taken at a high magnification, bright spots at the end of short grey lines can be seen on the sidewalls of isolated tubes or on external tubes of the bundles. A second less bright spot is systematically seen at the other end of these objects. The length of this kind of object was systematically measured between 1.2 and 1.4 nm, which agrees with the presence of a FcETG_2_ grafted group. Figure 10 illustrates this fact by presenting typical micrographs of high-resolution STEM images recorded on the HIPCO-HNO_3_-FcETG_2_ sample. In the HAADF micrograph (Figure 10a) the arrows point to the two sides of the typical object. The structure of the CNT that is present at the outer edge of a bundle was determined from the fast Fourier transform (FFT) of the BF image presented in Figure 10b. The chiral indices (11,5) can be unambiguously assigned to this tube.

To strengthen the interpretation of the images obtained on the small object lying at the surface of the tube, image simulations were done for a SWCNT grafted with a FcETG_2_ group. A structural model of a SWCNT with (11,6) chiral indices was used for the simulation (structure and diameter close to that of (11,5)). The FcETG_2_ molecule grafted at the surface of the SWCNT model was oriented to simulate a ferrocene group in a π-stacking interaction (see below the molecular dynamics simulation) since the analysis is done in ultra-high vacuum. The distance between the tube and the cyclopentadiene was fixed at 0.35 nm. Simulations were carried out in steps of 1 nm for focuses from −12 nm to 12 nm for the HAADF and BF detectors. For HAADF simulations the contrast obtained matches well with that experimentally detected (Figure 10c and Figure 10d, compared to the inset of Figure 10a). The bright dot corresponds to the ferrocene group, the gray line to the polyethylene glycol linker, and the less bright dot at the other extremity to the ester link between the CNT and the linker. The contrast obtained is not sensitive to the focus (in the focus range used). For BF images, the contrast obtained depends strongly on the focus and contrast inversion can occur as seen in Figure 10d and Figure 10f.

The image simulation can be used to estimate the focus of the images by comparing the recorded image to simulations for different focuses. For example the focus of the BF image presented in Figure 10b was estimated slightly over-focused by 1 or 2 nm. Numerous STEM images were analyzed for different samples, and the functionalization of the sidewalls was observed for both metallic and semiconducting tubes oxidized with either HNO_3_ or H_2_SO_4_ acidic conditions. Therefore, these results strongly support the hypothesis that outer tubes of the bundles were covalently grafted with FcETGn groups.

As a conclusion to this analytical part, we have succeeded in covalently grafting ferrocene derivatives on HIPCO SWCNTs, to a sufficiently small extent that we have preserved the structural integrity of the nanotubes. The complete set of data is consistent with the oxidation of pre-existing defects under microwave irradiation in acidic media. Isolated CNTs or those constituting the outer tubes of the bundles are functionalized covalently by ferrocene derivatives on their sidewalls. Both acidic conditions give similar results. The diluted H_2_SO_4_ condition is therefore quite interesting since it avoids the use of a concentrated acid solution. Using H_2_SO_4_ with microwave irradiation therefore represents a fast, green, safe and energy efficient process if one is interested in development on a semi-industrial scale.

These functionalized samples were then used to modify a GCE in the bioelectrochemical device presented in Figure 1, to test their capability in electrochemical devices.

### Electrochemical measurements

**Figure 11:**
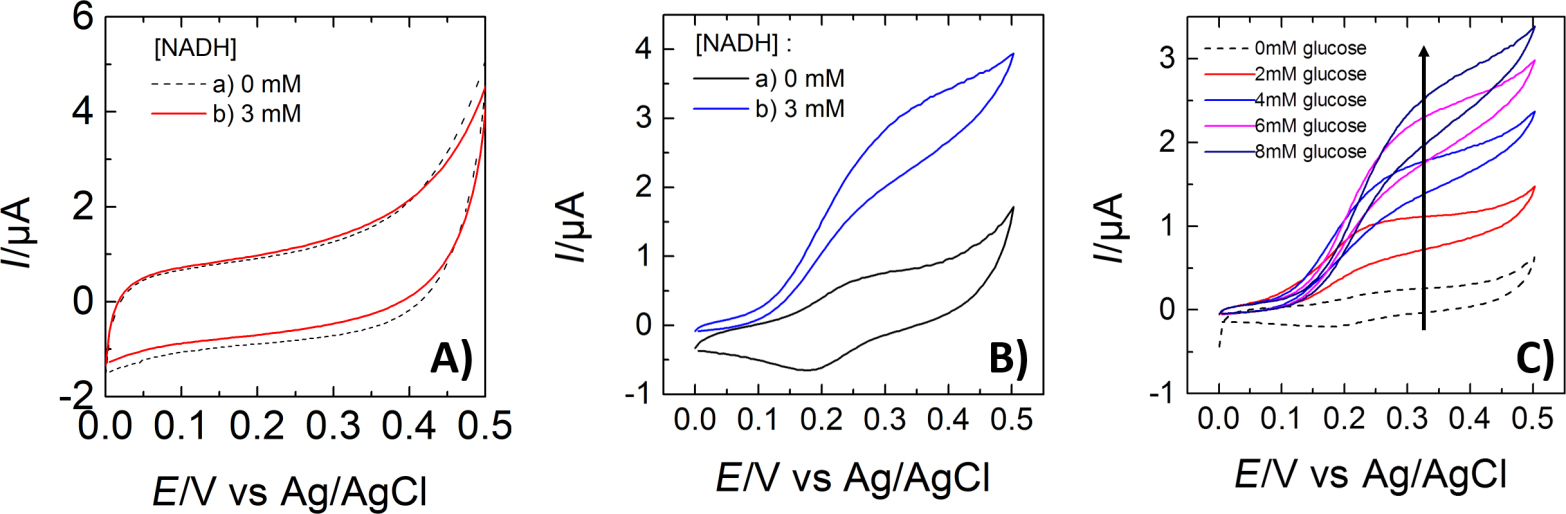
Cyclic voltammograms obtained of GCEs modified with A) HIPCO-H_2_SO_4_ immobilized with diaphorase; B) HIPCO-H_2_SO_4_-FcETG_2_ immobilized with diaphorase; C) HIPCO-H_2_SO_4_-FcETG_2_ immobilized with diaphorase, NADH and glucose dehydrogenase (GDH) molecules. In A and B, NADH was added to the analyzed solution; in C, glucose was added to the solution. The modified GCE electrodes were used as working electrodes in a conventional three-electrode setup. The scan rate was 5 mV·s^−1^. Arrows in B indicate the anodic and cathodic wave of the grafted ferrocene groups.

The electrochemical response of oxidized SWCNTs, i.e., before their functionalization with Fc, was tested first. The electrode was prepared by dispersing the carbon material in a 0.5 wt % chitosan solution. A layer of this chitosan composite was deposited on the GCE and a second layer of chitosan containing diaphorase was additionally deposited on the top. The GCE was used as the working electrode in a conventional three-electrode setup (see Figure S4 in Supporting Information File 1). Diaphorase can catalyze the oxidation of NADH but only in the presence of a mediator (electron shuttle) transferring electrons from the flavin mononucleotide cofactor to the electrode surface. Figure 11A reports the electrochemical response of the oxidized SWCNTs in the absence and in the presence of 3 mM of NADH in the solution. Both curves are very similar, characterized by a large capacitive current (due to the high surface area of the electrode modified with SWCNTs). The lack of any current increase after addition of NADH in solution indicates that these oxidized clean HIPCO SWCNTs do not exhibit any direct electrocatalytic properties toward NADH oxidation.

While previous reports attributed the catalytic properties of CNTs mainly to their impurities [21–26], the small amount of residual impurities in our HIPCO samples are not electroactive. Oxidized CNTs were used as a blank sample (Figure 11A) in order to verify that the oxidation process has not activated residual impurities nor generated electroactive groups such as quinones [6] at the CNT surface. This was not the case in our experiments. Moreover, this experiment confirms that no direct electron transfer can be achieved between diaphorase and the electrode, even in the presence of SWCNTs.

The functionalization of SWCNTs with ferrocene groups allowed for observing by cyclic voltammetry a quasi-reversible electrochemical signal at +0.215 V vs Ag/AgCl as is illustrated in curve a) of Figure 11B for sample HIPCO-H_2_SO_4_-FcETG_2_. All samples functionalized with Fc showed this pair of redox peaks. Some variation in capacitive current was observed between the different samples that must be related to the ease of f-SWCNT dispersion and the resulting different 3D textures after their immobilization at the GCE surface. For example, it was found that the H_2_SO_4_ oxidation process produced a more porous SWCNT assembly, and long polyethylene glycol linker (*n* > 4) favored dispersion of the nanotubes in water-based solutions. In spite of these differences, all oxidation processes and FcETG*_n_* linkers gave the kind of curves reported in Figure 11B.

Cyclic voltammetry was then performed with f-SWCNTs in the presence of NADH (curve b) in Figure 11B. The electrochemical response was dramatically modified, with the disappearance of the cathodic peak and the large increase in intensity of the anodic peak. This response is due to the electrocatalytic oxidation of NADH by diaphorase, during which the flavin mononucleotide cofactor is oxidized by ferrocenium produced at the electrode surface. No clear trend was observed that could have related the mode of oxidation (HNO_3_ or H_2_SO_4_) or the linker size for the ETG*_n_* spacer with the electrocatalytic response. The electrochemical response was most probably controlled by the availability of Fc on the surface, which mainly depended on the dispersion of the tubes in the chitosan layer. Previous analyses of the electrocatalytic properties of f-SWCNTs performed with less pure SWCNTs have shown that the size of the linker does not influence the NADH electrocatalytic current [11]. Contrary to these conclusions with polyethylene glycol linkers, functionalization with alkyl-ferrocene only showed a very poor electrocatalytic response (Figure S5 in Supporting Information File 1). The alkyl spacer certainly favored the folding of the chain in an aqueous solution and prevented the ferrocene group from efficiently exchanging electrons with diaphorase molecules.

In order to illustrate an application of these f-SWCNTs in biosensing, the electrode was further modified with HIPCO-H_2_SO_4_-FcEtG_2_, diaphorase, NADH and glucose dehydrogenase (GDH). Upon addition of glucose into the solution, the molecules were oxidized by GDH in gluconolactone and NADH cofactor was simultaneously produced. NADH was then oxidized by diaphorase, mediated by ferrocene moieties. Figure 11C shows the electrochemical response in the absence of glucose (dashed curve, 0 mM of glucose). The reversible electrochemical signal of ferrocene can be distinguished as in curve a) of Figure 11B. After addition of glucose, the electrochemical current increases dramatically, showing again the typical shape of an electrocatalytic response.

Electrochemical characterization by variation of the potential scan rate shows moreover that the current response varied linearly with the scan rate (Figure S6 in Supporting Information File 1). This means that the electrochemical process is confined at the surface, not determined by diffusion of ferrocene into the solution. So the mobility of ferrocene is just sufficient for efficient mediation of the electron flow from the flavin mononuclotide cofactor to the electrode surface.

The fact that the alkyl linker is completely inefficient for the mediated electron transfer process, while the polyethylene glycol spacers are efficient regardless of their length (in the range examined in this work) was puzzling, so we decided to investigate the role of the linker by molecular dynamics computation. The samples were used in an aqueous medium. The alkyl linker being hydrophobic while PEG linkers are hydrophilic strongly suggests also modelling the interaction between the functionalized nanotubes and the water molecules. The corresponding results are reported in the next paragraph.

### Determining the role of the PEG linker via molecular dynamics calculations

**Figure 12:**
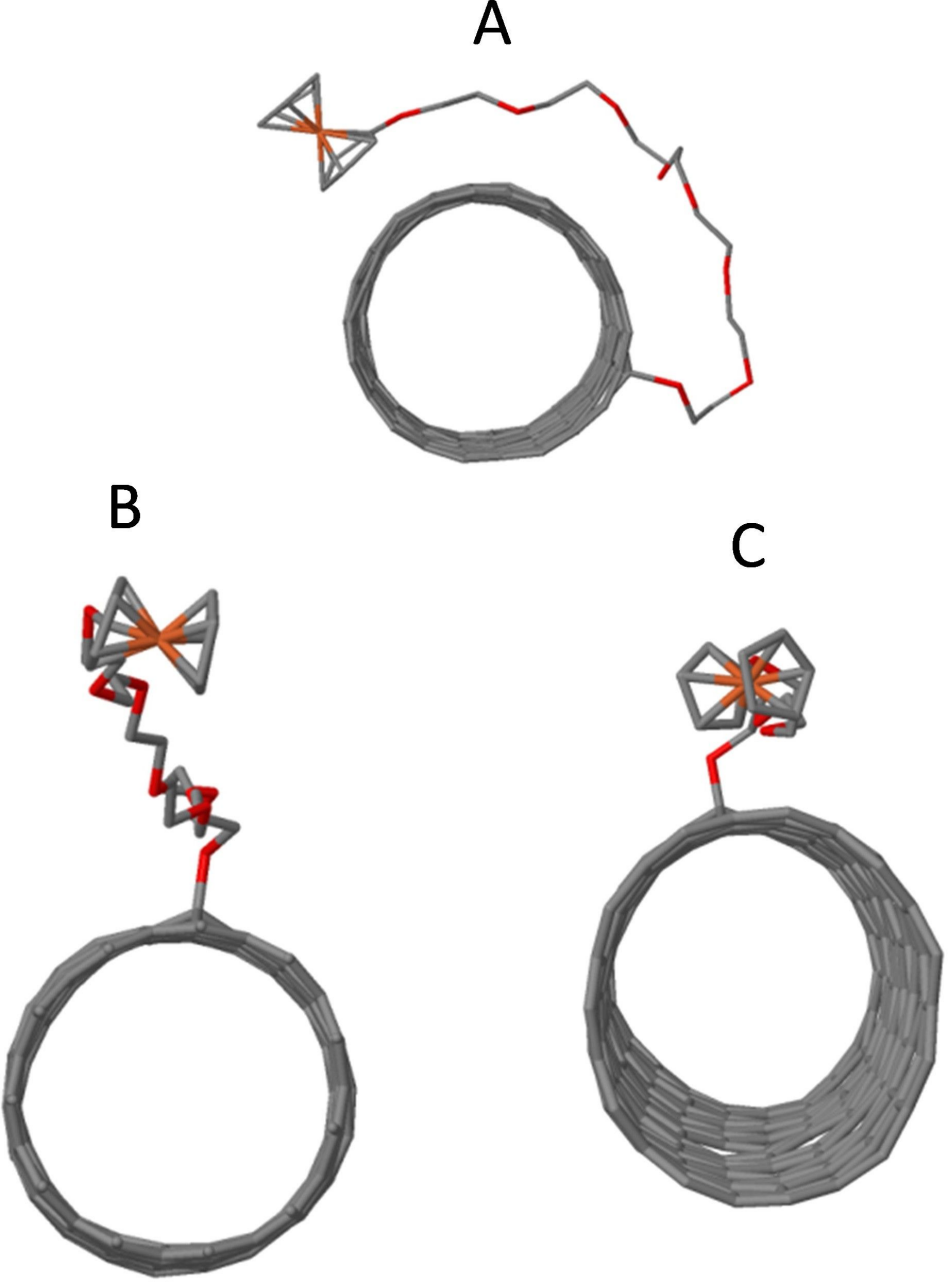
Molecular-dynamics simulated structures after 6 ns: A) HIPCO-FcETG_8_ without the presence of water, B) HIPCO-FcETG_8_ and C) HIPCO-FcETG_2_ in the presence of water (not shown for clarity).

The influence of the linker length on the resultant enzymatic reactivity of the ferrocene unit was explored using the molecular dynamics package LAMMPS [32–33] to simulate both FcETG_2_ and FcETG_8_ when covalently attached to the surface of a (10,0) carbon nanotube (radius typical for HIPCO samples). A tube length of 4.17 nm was chosen (440 carbon atoms) placed in a cubic unit cell of the same length creating an infinite tube in one direction and enough “vacuum” for the PEG chain to relax without constraint. The calculations were performed using a ReaxFF potential [34]. The runs were carried out for 6 ns with a time step of 0.1 fs. Time integration (10 fs) was used on Nosé–Hoover [35–36] style non-Hamiltonian equations of motion, designed to generate positions and velocities sampled from the canonical (npt) method (constant pressure and temperature). All calculations were carried out at room temperature and under atmospheric pressure. Calculations were done both with and without the presence of water (1967 water molecules distributed uniformly initially within the box, giving a net water density of 1.01 g·cm^−3^ without HIPCO-FcETG_8_ and around 0.800 g·cm^−3^ with HIPCO-FcETG_8_). Any water molecule within a distance of less than 3.0 Å from the tube was removed (Figure 12).

In the absence of water, with both HIPCO-FcETG_8_ (Figure 12A) and HIPCO-FcETG_2_ after only 2 ns the ETG chain flexed to go around the tube following a zig-zag path on the CNT surface, allowing the ferrocene unit to π-stack on the nanotube where it remained for the rest of the simulation. We used this very useful information in our HRSTEM simulation in vacuum by placing the ferrocene unit in a π-stacking with the tube, in the results shown in Figure 10c–f. In such a configuration, a ferrocene electron transfer may occur between the ferrocene unit and the CNT surface instead of interacting freely with the diaphorase biomolecule. As a result, this would quench the enzymatic activity of the modified GCE. To explain why and how our device can work, we then simulated the effect of water.

Once water was added, van der Waals and dipolar interactions between the water molecules and the ETG chain effectively blocked chain flexion and maintained the ferrocene unit at a distance of over 6.41 Å from the tube surface throughout the simulation (Figure 12B). Even for HIPCO-FcETG_2_, while the water–ETG interactions are considerably reduced, which allowed for some chain flexion and more interaction with the surface (Figure 12C), the ferrocene unit is not π-stacked on the CNT surface and therefore the electron-transfer step with diaphorase is still possible. The simulations confirm the essential role of the PEG linker in the efficiency of the bioelectrochemical device in water, due to the favorable interaction between the ETG units and water molecules, which prevents π-stacking of the ferrocene unit on the surface of the CNTs.

## Conclusion

This work has clearly established the interest of using covalently functionalized SWCNTs for making electrochemical devices. Using a very clean sample, a complementary set of analytical techniques, including advanced microscopy analyses, has firmly confirmed the covalent nature of the functionalization process. We have shown that residual impurities in the case of mediated electron transfer play no role in the oxidation of NADH, contrary to what has been presented in the literature for other systems. We also underline the interest of using molecular dynamics simulations to evaluate the important role of the linker, which certainly depends on the solvent used for doing electrochemistry. The results of the present study therefore lead us to claim that, while many people are now working with graphene, CNTs are indeed useful for well-designed electrocatalytic devices, thus being a viable alternative to graphene-based sensors provided that the samples used are sufficiently clean so that the function is not controlled by the presence of residual impurities that may lead to unreproducible measurements.

## Experimental

**General:** COCl-SWCNTs were prepared according to [27]. FcETG*_n_* derivatives were synthesized according to [29].

**Reaction of COCl-SWCNTs with FcETGn** [9]**:** COCl-SWCNTs (10 mg) were suspended in dry toluene (50 mL) under argon. Then, FcETG*_n_* (20–30 mg) and triethylamine (0.1 mL) were added. The mixture was heated to 100 °C and stirred for 24 h under argon. After cooling, the SWCNTs were washed under sonication three times with MeOH (10 mL) and three times with THF (10 mL) in order to remove unreacted FcETGn. After filtration, the HIPCO-Ox-FcETG*_n_* (22–33 mg) were dried under vacuum and stored under argon at room temperature.

**Electrode preparation** [11]**:** GCEs (3 mm in diameter) were polished with alumina slurry (1 μm and 0.05 µm particles, sequentially) and then washed with water. A 0.5 wt % chitosan solution was prepared by dissolving chitosan in 1% acetic acid. 2 mg of ferrocene-functionalized CNTs (HIPCO-Fc) and 1 mL of the above chitosan solution were mixed together under stirring overnight. 5 µL of chitosan/HIPCO-Fc suspension were deposited onto the GCE surface to obtain a stable thin film after drying. Chitosan films containing either diaphorase (DI) or glucose dehydrogenase (GDH) and DI were prepared by mixing 10 µL of 0.5 wt % chitosan solution with 5 µL of DI (5 mg·mL^−1^) or 15 µL of 0.5 wt % chitosan with 10 µL of GDH (1000 U·mL^−1^) and 5 µL of DI (5 mg·mL^−1^). Afterwards, 5 µL of this mixture were deposited onto the chitosan/HIPCO-Fc modified GCE and allowed to dry at ambient temperature.

**Analytical techniques:** High-resolution transmission electron microscopy (HRTEM) and high-resolution scanning transmission electron microscopy (HRSTEM) were performed using a probe aberration-corrected JEOL ARM200 microscope equipped with a cold field emission gun, a JEOL JED2300T energy dispersive X-ray spectrometer (EDS) and a Gatan GIF Quantum energy filter for electron energy loss spectroscopy (EELS). The microscope was operated at 80 kV in order to minimize knock-on damage to the samples. HRSTEM images were recorded using an electron beam with a probe-size of about 0.12 nm. High-angle annular dark-field (HAADF) and bright-field (BF) images were simultaneously recorded with a semi-angle of collection of respectively 45–180 mrad and 11 mrad for an image resolution of 1024 × 1024 pixels with a dwell-time of 60 µs. STEM-EDS and STEM-EELS experiments were performed with an electron probe of about 0.17 nm and a convergence semi-angle of 24 mrad. The energy resolution was about 0.45 eV. The collection angle of the EELS spectrometer was 30 mrad. The spectroscopic information was obtained using the spectrum-imaging acquisition mode. Samples were dispersed in absolute ethanol then deposited on a holey carbon film supported by a 300 mesh copper grid. The STEM image simulations were done using the software Nanotube Modeler (JCrystalSoft) and Vesta 3 for the atomic model generation [37] and QSTEM [38] for the STEM image simulations. SWCNT chiral indices were assigned by analysis of the Fourier-transform of the HRSTEM images and by comparing the images to simulations [39–40].

Raman spectra were recorded with a triple subtractive monochromator T64000 spectrometer (Horiba Jobin Yvon) equipped with a confocal microscope (Olympus BH2-UMA; laser wavelengths λ = 514 nm and λ = 458 nm). An 80× objective with a numerical aperture of 0.90 was used to collect the spectra in the backscattering mode. The spectral resolution was 1 cm^−1^. The laser spot had a diameter of 1 µm and the irradiance was kept below 1 kW·cm^−2^ to avoid laser heating of the samples. At least 20 spectra were collected and averaged for each sample to obtain a statistically representative spectrum.

The XPS analysis was performed with a spectrometer equipped with a monochromatized Al Kα X-ray source (hν = 1486.6 eV), run at 150 W power (KRATOS Axis Ultra, Kratos Analytical, UK). Spectra were collected at a normal take-off angle (90°). The analytical area was 700 × 300 μm^2^. XPS survey spectra were recorded with a 1.0 eV step and 160 eV analyzer pass energy. Narrow scans were recorded with a 0.05 eV step and 20 eV pass energy. XPS spectra were analyzed with the Vision software from Kratos (Vision 2.2.0) after subtraction of the background (Shirley baseline). Gaussian–Lorentzian (70/30) functions were used for peak decomposition. Charge correction was carried out using the C 1s core line and setting adventitious carbon signal (C sp^3^ signal) to 284.6 eV.

For thermogravimetric analyses coupled to mass spectrometry (TGA-MS) experiments, 5–6 mg of the sample powder were placed in an alumina crucible. The TGA-MS instrument included a Setaram Setsys evolution 1750 Thermal Gravimetric Analyser coupled with a Pfeiffer GSD 301C Vacuum OmniStar mass spectrometer. The temperature was ramped at 3 °C/min from room temperature to 1000 °C under helium (flux 20 mL·min^−1^) in the TGA chamber. We consider that most of the species undergo one ionization only meaning that *z* = 1 for the detected *m*/*z* values.

All electrochemical experiments were carried out using a PGSTAT12 Metrohm-Autolab potentiostat monitored by the GPES Software. Measurements were performed in a three-electrode cell, including the film-modified GCE as working electrode, an Ag/AgCl reference electrode (3 M KCl internal electrolyte), and a platinum wire auxiliary electrode.

## Supporting Information

Includes XPS Fe 2p spectrum for HiPCO-H_2_SO_4_-FcETG_2_, supplemental TGA-MS and Raman data, a cartoon of the complete bioelectrochemical device, and additional electrochemical data.

File 1 Additional experimental data.
