# Supplementary material for: Accurate control of the covalent functionalization of single-walled carbon nanotubes for the electro-enzymatically controlled oxidation of biomolecules
Source: Beilstein J Nanotechnol. 2018 Oct 26;9:2750–62. doi: 10.3762/bjnano.9.257 (PMC6204796; doi:10.3762/bjnano.9.257)

Supporting Information

for

Accurate control of the covalent functionalization of single-walled carbon nanotubes for the electro-enzymatically controlled oxidation of biomolecules

Naoual Allali[1,2,3], Veronika Urbanova[1], Mathieu Etienne[1], Xavier Devaux[4], Martine Mallet[1], Brigitte Vigolo[5], Jean-Joseph Adjizian[6], Chris P. Ewels[6], Sven Oberg[3], Alexander V. Soldatov[3], Edward McRae[5], Yves Fort[2], Manuel Dossot*[1] and Victor Mamane*[2,7]

Address: [1]LCPME, UMR CNRS-Université de Lorraine 7564, 405 rue de Vandoeuvre, F-54602 Villers-lès-Nancy, France; [2]SRSMC, UMR CNRS-Université de Lorraine 7565, Campus Victor Grignard, Faculté des Sciences et Technologies, F-54506 Vandoeuvre-lès-Nancy, France; [3]Department of Engineering Sciences and Mathematics, Lulea Technical University, Sweden; [4]IJL, UMR CNRS-Université de Lorraine 7198, Parc de Saurupt - CS 50840, 54011 Nancy Cedex, France; [5]IJL, UMR CNRS-Université de Lorraine 7198, Campus Victor Grignard, Faculté des Sciences et Technologies, 54506 Vandoeuvre-lès-Nancy Cedex, France; [6]IMN, UMR CNRS-Université de Nantes 6502, 2 Rue de la Houssiniere, BP32229, 44322 Nantes Cedex, France and [7]New address: Institut de Chimie de Strasbourg, UMR CNRS-Université de Strasbourg 7177, 1 rue Blaise Pascal, F-67000 Strasbourg, France

Email: Manuel Dossot - manuel.dossot@univ-lorraine.fr; Victor Mamane - vmamane@unistra.fr

* Corresponding author

Additional experimental data

Figure S1A. Global XPS survey spectra of raw HIPCO and HIPCO-H_2SO_4-$FcETG_8$ samples deposited on gold surfaces

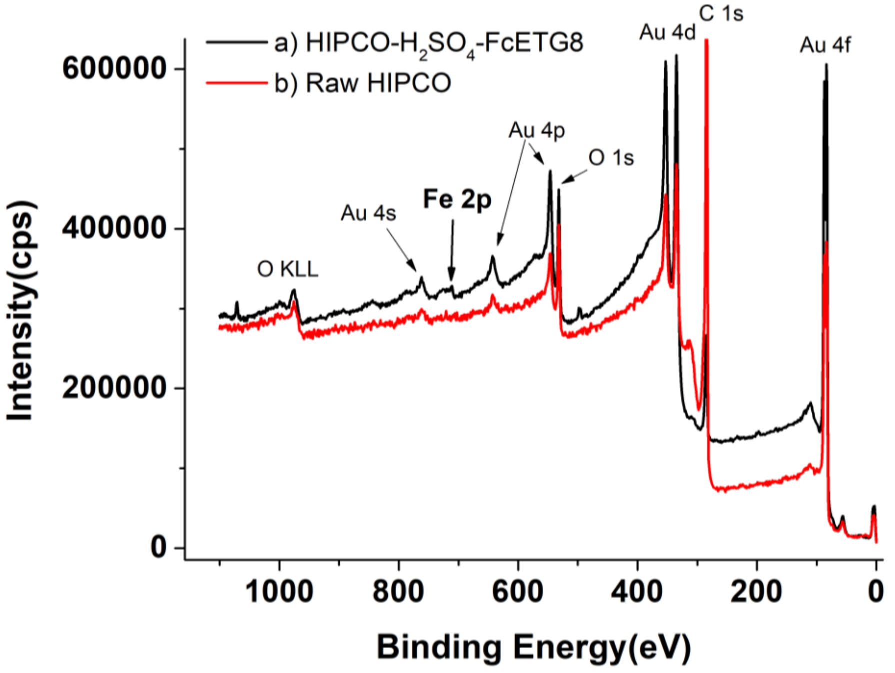

Figure S1B. Detailed XPS spectra in the Fe 2p region for the HiPCO-H_2SO_4-$FcETG_2$ sample

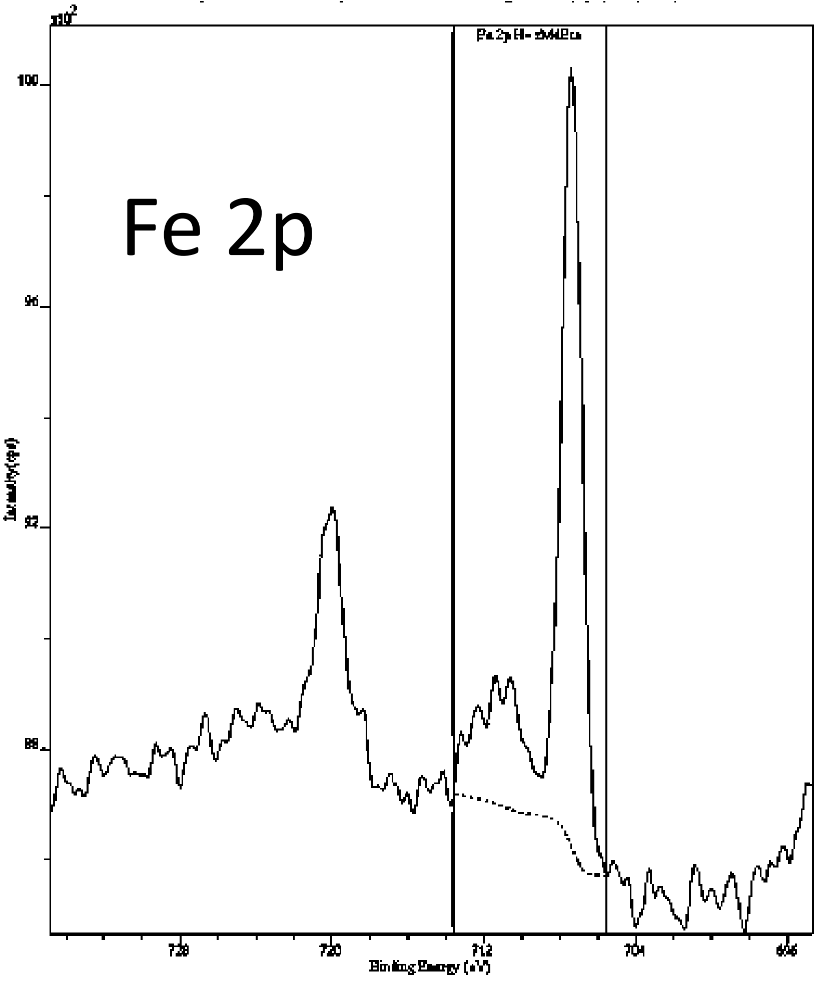

Figure S2. TGA-MS detection of CO and CO2 fragments for A) HIPCO-HNO_3 and B) HIPCO-HNO_3-FcETG$_2$ samples.

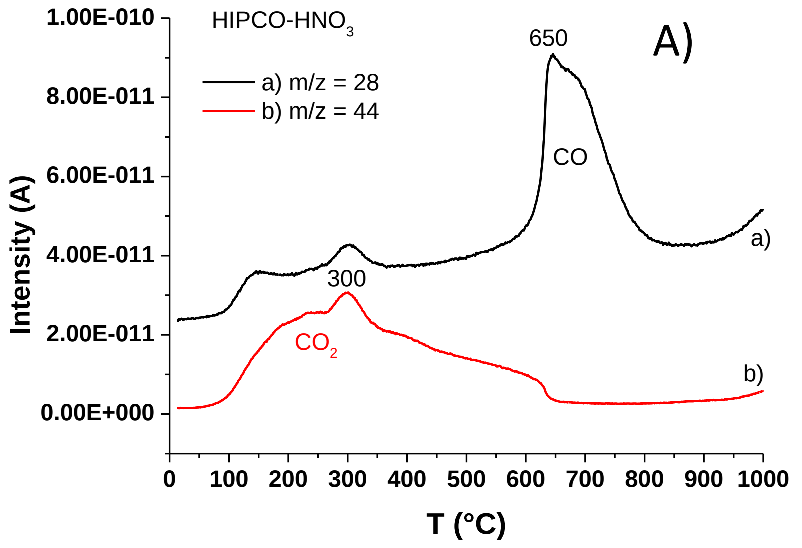

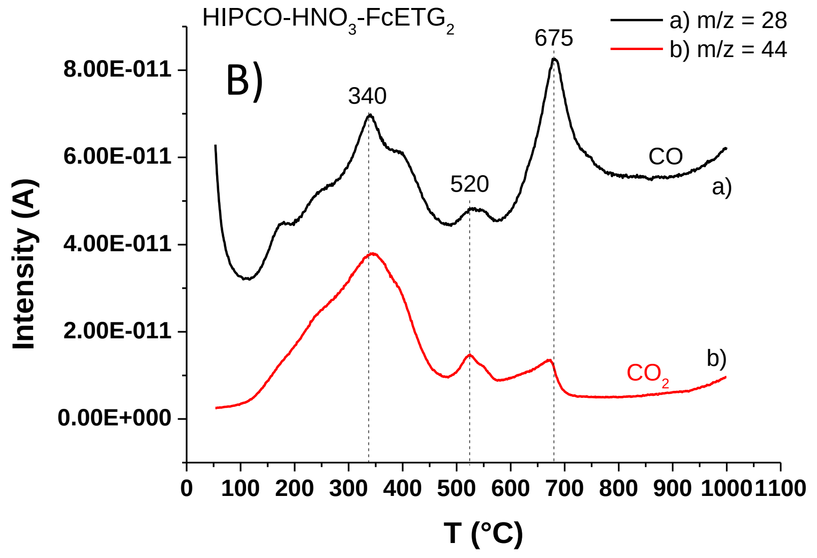

Figure S3. Raman spectra taken using a laser wavelength of 458 nm for samples oxidized by HNO_3 and functionalized with ferrocene derivatives.

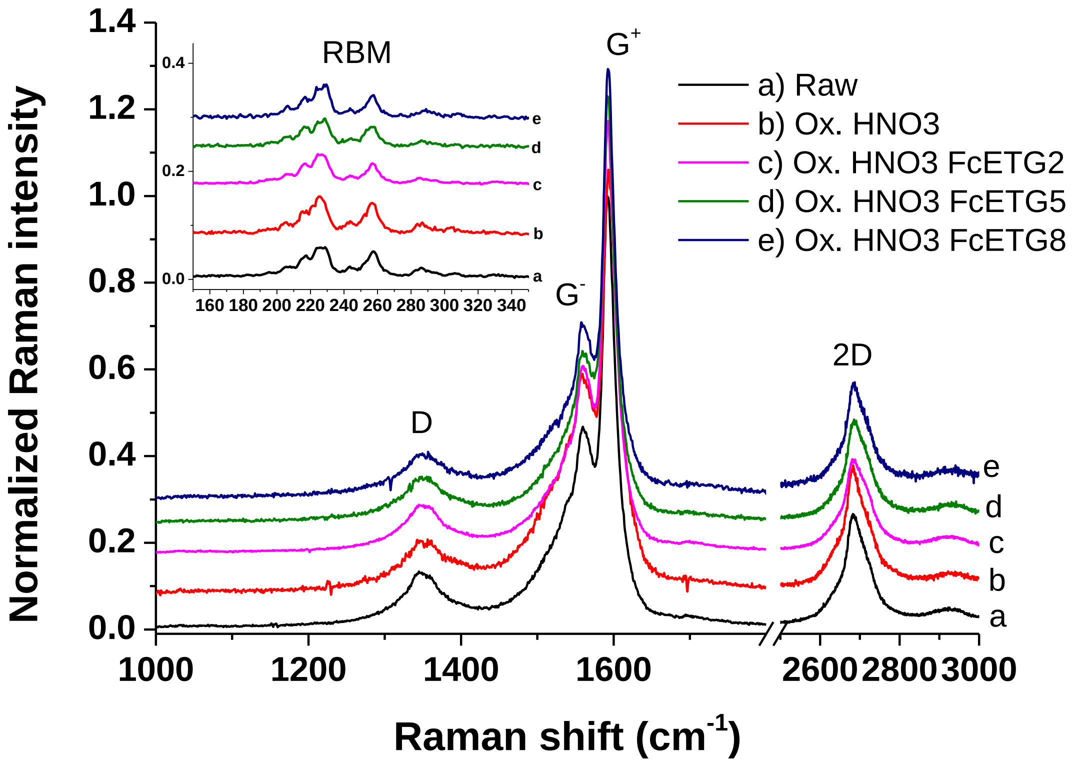

Figure S4. A) Incorporation of the modified GCE in a three-electrode set-up; B) principle of the complete biosensor for glucose detection.

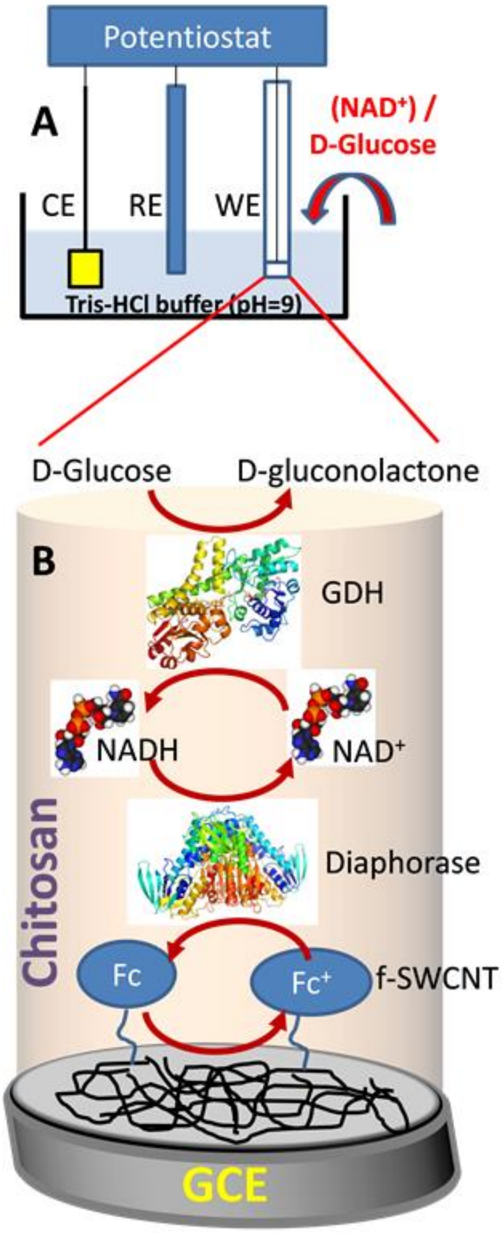

Figure S5. Cyclovoltammogram of HIPCO-HNO_3-FcAlkyl

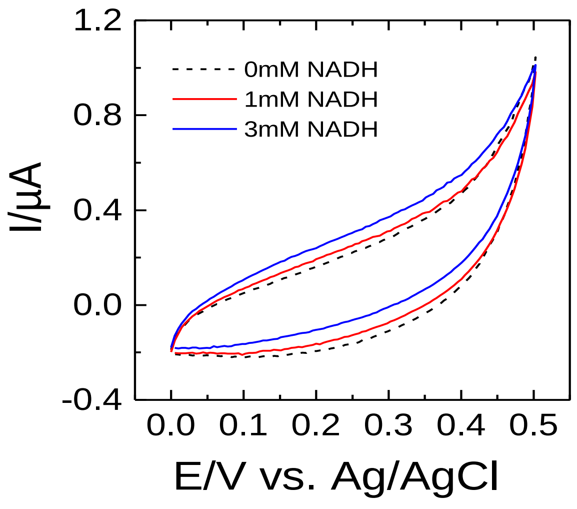

Figure S6. A) Variation of the cyclovoltammogram of HIPCO-H_2SO_4-FcETG$_2$ samples with the scan rate; B) Current intensities of the anodic and cathodic peaks as a function of the scan rate.

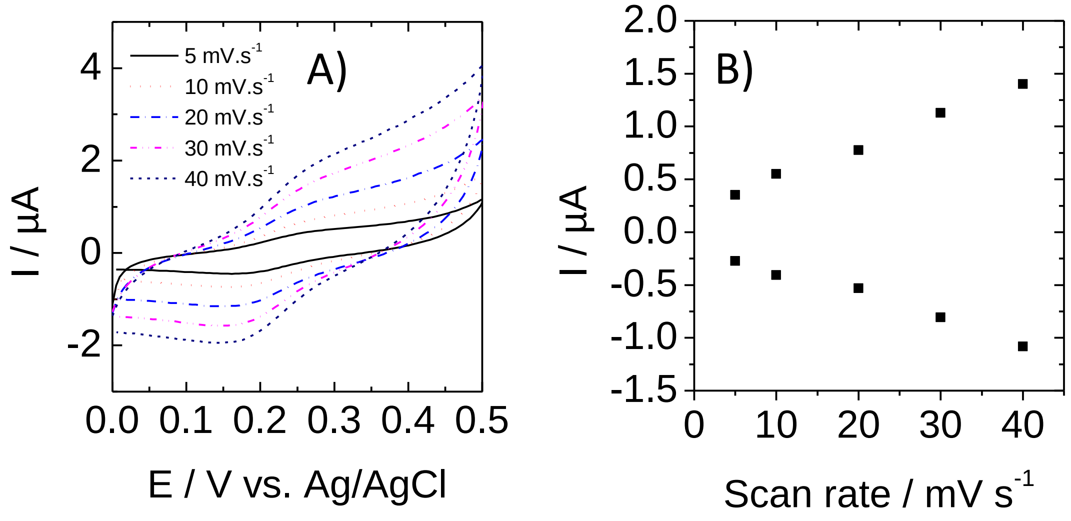

Supplement: File 1 — Additional experimental data. [file Beilstein_J_Nanotechnol-09-2750-s001.pdf]
